# Supplementary material for: Identification and functional analysis of non-coding regulatory small RNA FenSr3 in Bacillus amyloliquefaciens LPB-18
Source: PeerJ. 2023 May 15;11:e15236. doi: 10.7717/peerj.15236 (PMC10194069; doi:10.7717/peerj.15236)
Supplement: Supplemental Information 4 [file peerj-11-15236-s004.zip › KO/CK-vs-T1_map/map04920.html]

KEGG PATHWAY: Adipocytokine signaling pathway - Reference pathway


|  |  |
| --- | --- |
| **Adipocytokine signaling pathway - Reference pathway** |  |

[
Pathway menu
| Organism menu
| Pathway entry
| Show description
| User data mapping
]

Increased adipocyte volume and number are positively correlated with leptin production, and negatively correlated with production of adiponectin.
Leptin is an important regulator of energy intake and metabolic rate primarily by acting at hypothalamic nuclei. Leptin exerts its anorectic effects by modulating the levels of neuropeptides such as NPY, AGRP, and alpha-MSH. This leptin action is through the JAK kinase, STAT3 phosphorylation, and nuclear transcriptional effect.
Adiponectin lowers plasma glucose and FFAs. These effects are partly accounted for by adiponectin-induced AMPK activation, which in turn stimulates skeletal muscle fatty acid oxidation and glucose uptake. Furthermore, activation of AMPK by adiponectin suppresses endogenous glucose production, concomitantly with inhibition of PEPCK and G6Pase expression.
The proinflammatory cytokine TNFalpha has been implicated as a link between obesity and insulin resistance. TNFalpha interferes with early steps of insulin signaling. Several data have shown that TNFalpha inhibits IRS1 tyrosine phosphorylation by promoting its serine phosphorylation. Among the serine/threonine kinases activated by TNFalpha, JNK, mTOR and IKK have been shown to be involved in this phosphorylation.

|  |  |  |
| --- | --- | --- |
| Reference pathway | 184% 150% 122% 100% 82% 67% 55% | 图片下载 |
